# Maximum parsimony interpretation of chromatin capture experiments

**DOI:** 10.1371/journal.pone.0225578

**Published:** 2019-11-25

**Authors:** Dirar Homouz, Andrzej S. Kudlicki

**Affiliations:** 1 Department of Physics, Khalifa University of Science and Technology, Abu Dhabi, UAE; 2 Department of Physics, University of Houston, Houston, TX, United States of America; 3 Center for Theoretical Biological Physics, Rice University, Houston, TX, United States of America; 4 Institute for Translational Sciences, University of Texas Medical Branch, Galveston, TX, United States of America; 5 Department of Biochemistry and Molecular Biology, University of Texas Medical Branch, Galveston, TX, United States of America; University of Crete & IMBB-FORTH, GREECE

## Abstract

We present a new approach to characterizing the global geometric state of chromatin from HiC data. Chromatin conformation capture techniques (3C, and its variants: 4C, 5C, HiC, etc.) probe the spatial structure of the genome by identifying physical contacts between genomic loci within the nuclear space. In whole-genome conformation capture (HiC) experiments, the signal can be interpreted as spatial proximity between genomic loci and physical distances can be estimated from the data. However, observed spatial proximity signal does not directly translate into persistent contacts within the nuclear space. Attempts to infer a single conformation of the genome within the nuclear space lead to internal geometric inconsistencies, notoriously violating the triangle inequality. These inconsistencies have been attributed to the stochastic nature of chromatin conformation or to experimental artifacts. Here we demonstrate that it can be explained by a mixture of cells, each in one of only several conformational states, contained in the sample. We have developed and implemented a graph-theoretic approach that identifies the properties of such postulated subpopulations. We show that the geometrical conflicts in a standard yeast HiC dataset, can be explained by only a small number of homogeneous populations of cells (4 populations are sufficient to reconcile 95,000 most prominent impossible triangles, 8 populations can explain 375,000 top geometric conflicts). Finally, we analyze the functional annotations of genes differentially interacting between the populations, suggesting that each inferred subpopulation may be involved in a functionally different transcriptional program.

## Introduction

The three-dimensional organization of eukaryotic genome inside the nuclear space has been shown to play an important role in the regulation of transcription. In the last decade, our understanding of the genome organization has greatly progressed thanks to experimental techniques such as chromosome conformation capture (3C), 4C, 5C, 6C, ChIA-PET, and HiC [1–6]. In these methods, three-dimensional contacts between different parts of the DNA are captured by ligation, and characterized, typically by sequencing and mapping to their genomic loci. The contacts provide information on the spatial organization of the genome. Recent developments in these technologies have allowed high-resolution mapping of the interactions within in the entire genome.

The spatial, Euclidean distances between interacting loci can be estimated from chromatin capture data [6, 7], and may, in turn, be used for constructing three-dimensional models of the entire genome [6]. Such models have been proposed mainly for illustrative purposes, as the chromosomes are thought to be highly dynamic [8–10], and the greatest value of HiC experiments is in detecting functional interactions between genomic loci. It remains nonetheless an open question how permanent the interactions are, how many degrees of freedom are realized in the configuration space of actual nuclei, and whether any differences between conformations of individual cells are caused by inherent differences between the cells or rather by the conformations constantly changing with time. To gain insight into these questions, we here characterize the geometric constraints on chromatin conformations derived from HiC data and infer certain properties of cell-to-cell variability.

We have observed that interpreting DNA interaction data as distances within a single conformation, may lead to impossible geometries. These inconsistencies arise when a global 3-D model is constructed of the whole genome, but also when locally relevant groups of interactions are considered. Specifically, in a haploid cell, a conformation is not possible in which *locus C* is close to *locus A* and to *locus B*, but the Euclidean distance between the loci *A* and *B* is large:

d(AB)≫d(AC)+d(BC), (Eq 1)

where d() denotes the physical, Euclidean distance between two loci in the nuclear space. Such case would lead to an impossible geometry, violating the triangle inequality. Such a situation corresponds to strong 3C signal for the AC and BC interactions, but few or no reads that would correspond to interaction between A and B. When using the standard formula to estimate the Euclidean distance between loci from HiC reads in the yeast HiC data of [11], we find large numbers of such impossible triangles. Observation of such apparently impossible geometries may be attributed to a range of possible causes, including inaccuracy of the formula, noise in the data, systematic errors such as sequencing bias, and others, including experimental errors [12]. Another possible explanation is that the chromatin conformation is not rigid but constantly changing and that the HiC experiments represent not one conformation but rather an entire ensemble of states accessible by small, thermal-like motions.

Such data-driven models implicitly assume that the population properties of cells used in the experiment are either uniform with respect to the chromatin conformation or occupy a relatively small, presumably connected, volume in the conformational space; however, this is not necessarily true, as it is known from fluorescent microscopy that the global chromatin structure may significantly vary between cells [13]. The inability to appreciate the dynamics and variability of chromatin states has been indicated as the main drawback of HiC based methods [14], triggering research into developing single-cell HiC experimental techniques. As stated above, the HiC data may be reconciled by an ensemble of dynamic, continuously changing conformations, or by one conformation with a specific pattern in measurement errors. In metazoans, numerical simulations of continuous, stochastic variability are consistent with certain characteristics of experimental measurements [15–17]. The objective of this paper is to demonstrate that yet another type of model may be possible that fully explains the experimental data. To this end, we take the ensemble proposition to the opposite extreme: rather than considering a continuous ensemble of possible chromatin conformations, we investigate whether it is possible that the measured HiC signal is produced by a small number of discrete, rigid conformations of DNA. We assess how many such conformations would be required, and what are their properties. To this end, we developed a graph-theoretic approach to this problem, that is presented below, along with and the resulting characterization of such postulated rigid states.

## Results

### Characterization of the geometric conflicts

As our primary dataset, we use the standard yeast data of Duan et al [11], providing DNA contact information with kilobase resolution for a model haploid genome. The experiment probed chromatin interactions for all pairs of HINDIII restriction enzyme target loci in the yeast genome. To convert read counts to approximate physical distances, we use the formula derived by [7]:

$$D=d_{0}/N.$$ (2)

The value of d_0_ has been estimated by [11] as 155,000 nanometers, however, in the present considerations the numeric value is never used in the calculations, it will cancel out as long as it remains approximately constant.

To characterize the conflicts, we introduce a working definition of the triangle inequality [Eq 3]. We call a triangle ABC (AB being the longest side) “*impossible”* when the estimated distances satisfy the following condition:

d(AB)>a*[d(AC)+d(BC)], (3)

for a given a > 1.

We applied Eq (3) to all triples of HINDIII loci in the HINDIII dataset of ([11]). As a result, for values of *a* from 1.3 to 2, we obtain between 30953 and 664101 conflicts (impossible triangles) in the genome that affect the majority of HINDIII sites in the genome (See column 2 and 3 of Table 1). It is important to note that we base this analysis only on the raw contact data from the HiC dataset of [11], but not on the approximate 3-D snapshot of the genome provided in that same paper.

**Table 1:** Characterization of geometric conflicts (violations of triangle inequality) for the yeast dataset of Duan et al, at different thresholds of conflict definition *a*.

*a*	Conflicts	HINDIII loci involved in conflicts	In coding sequence	within 500bp from TSS	HINDIII loci acting as mid loci	Mid loci in coding sequences	Mid loci within 500bp from TSS
1.3	664101	3476	1338	460	2810	1074	360
1.4	374735	3442	1323	455	2712	1032	348
1.7	95370	3375	1298	446	2326	889	295
2.0	30953	3247	1246	428	1834	700	228

**Fig 1:**
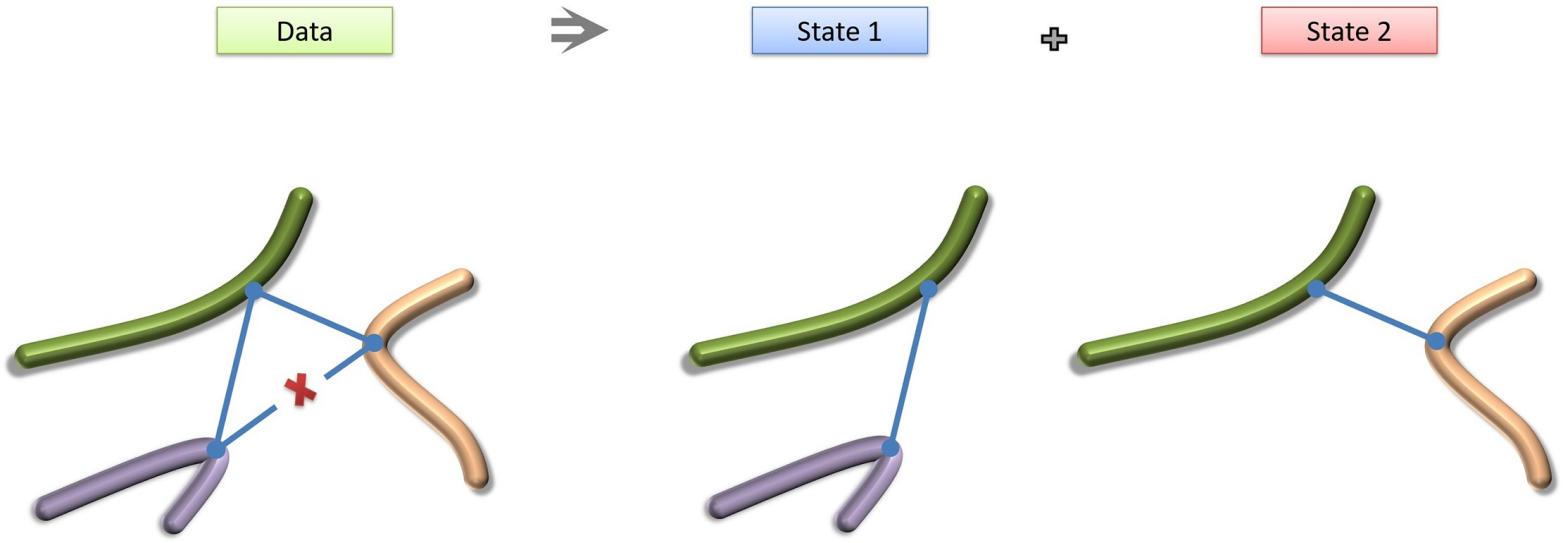
A schematic representation of three loci in a haploid genome, forming an impossible triangle. If tight DNA contacts (blue lines) are observed for two pairs of loci, but no signal is present for the third one (marked by red X), then the two observed interactions cannot coexist in one type of haploid cell and must represent distinct chromatin states.

Since the 3D structure of the genome is associated with transcriptional regulation, we next tested whether there is a dependence between HINDIII site involvement in conflicts and its association with coding sequence or transcription start sites. Since a vast majority of HINDIII loci in the yeast genome are involved in at least one of the geometrical conflicts, the global enrichments are not highly significant. An impossible triangle (as defined by Eq 1 and Fig 1) consists however of three HiC loci, the “promiscuous”, mid-site C that is in contact with both other two sites, and the “exclusive” A and B ends that show HiC signal only with C, but not with each other. While most HINDIII loci show some involvement in the conflicts, only a fraction of them assume the role of the promiscuous, “C” vertex of the triangle. Still, the fraction of “mid” C-loci to all loci in contacts does not change significantly when only loci near TSS, or loci in coding sequence are considered.

## Resolving the spatial conflicts

### The mixed-state hypothesis

The fact that a large number of geometric inconsistencies are present in HiC data makes it a crucial problem in interpreting chromatin capture 3C experiments and calls for identifying a solution to the issue. The existence of an impossible triangle may be explained by a range of factors, such as experimental error, or limited applicability of Eq (2). It is also possible that stochastic motions occasionally bring loci A and C or loci B and C together, but for some reasons loci A and B are never in close spatial proximity.

Here, we introduce an alternative hypothesis and verify that it is consistent with the data. We propose that the HiC data may result from a small number of discrete conformations of genomic DNA, each conformation corresponding to a specific subpopulation of cells within the experimental sample. An “impossible triangle” inferred from the data corresponds to two interactions (AC and BC) whose coexistence is ruled out due to lacking evidence of proximity between A and B. Our working hypothesis is rooted in the proposition that AC and BC indeed do not coexist in the same cells; as some cells have only the AC interaction while others only BC. The concept of disentangling the population-averaged measurements is presented graphically in Fig 1.

While resolving one geometrical conflict requires two distinct subpopulations of cells in the experimental sample, one might expect that the number of subpopulations needed to explain the thousands of conflicts in the data would be very large, suggesting that the approach is not practical at all. However, just one pair of subpopulations may be enough for explaining more conflicts. For example, if AC is incompatible with BC, and XZ cannot coexist with YZ, it is possible within our framework that AC and XZ exist in subpopulation I, while BC and YZ coexist in subpopulation II. Below we introduce and implement a graph-theoretic approach to determine and characterize the minimal number of subpopulations required to reconcile all the conflicts found in the data.

### Globally resolving conflicts in HiC contacts

**Fig 2:**
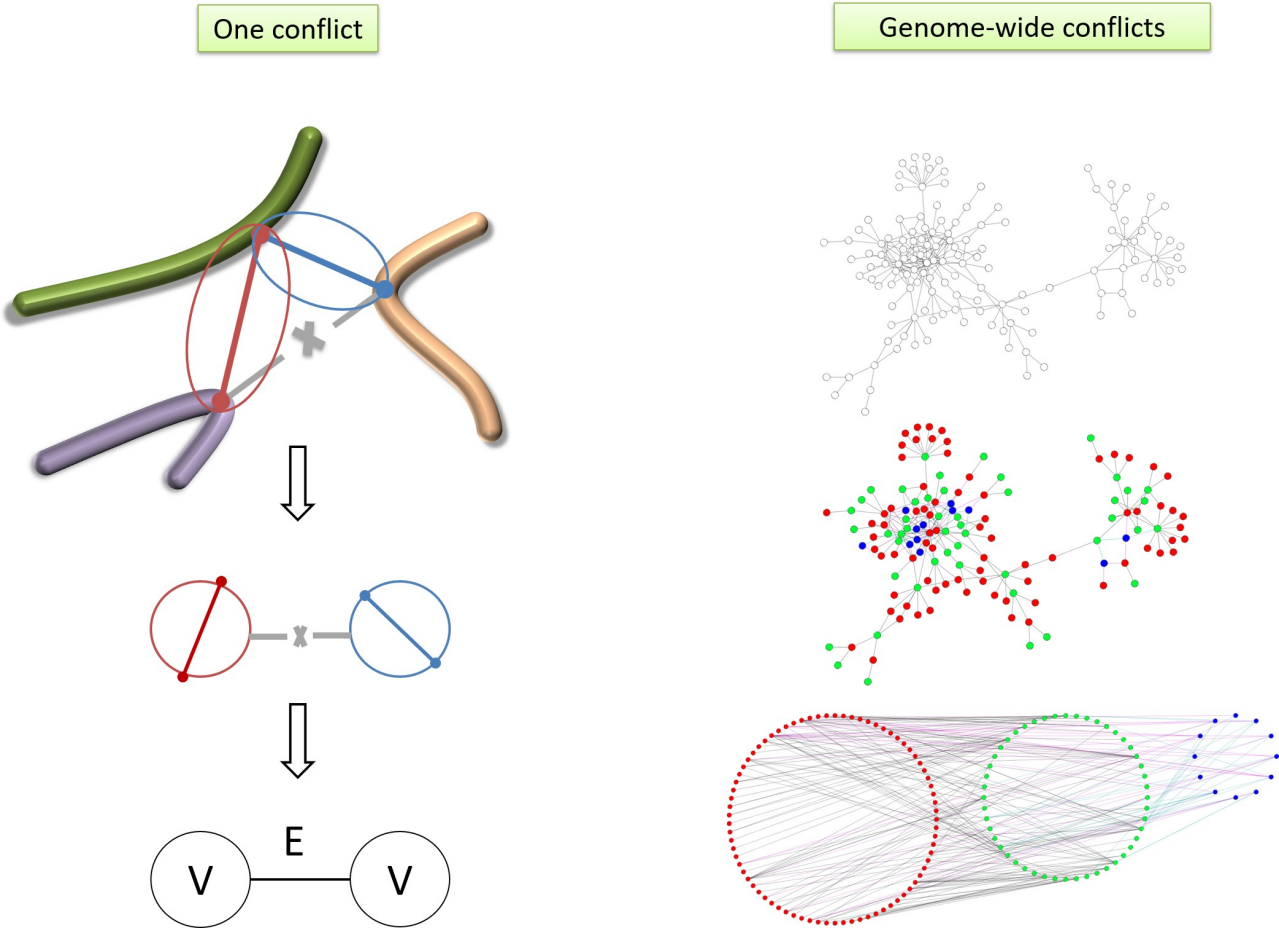
Resolving contact conflicts. Left: Generating the conflict graph, where the vertices are contacts and edges are conflicts. Right: Assuming a mixed population of cells in the sample, we resolve the conflict by coloring the (V,E) conflict graph.

The list of all observed conflicts is used to create a graph G = (V, E) where each vertex in V represents a contact (a pair of interacting sites; note that the graph is dual to a graph representing genomic loci as vertices and interactions as edges). Two vertices are connected by an edge in E if they are in conflict, that is the two interactions cannot coexist in the same homogeneous subpopulation of cells (Fig 2). The problem of finding the minimal number of subpopulations that can reconcile the experimental data is equivalent to coloring the vertices of the graph G such that two vertices with the same color must not be connected with an edge (two interactions occurring in the same subpopulation must not be incompatible due to forming an impossible triangle). This means that two conflicted contacts will be colored differently and thus always belong to different colors (or states of the genome). A graph-coloring algorithm will find the chromatic number of the graph, i.e. minimum number of colors for a graph and will color the vertices according to this minimum. Coloring of vertices of a graph is a classical problem in mathematics and computer science. The graph coloring algorithm we used is based on the column generation principle. The graph coloring algorithm that we used here is a modification of the heuristic approach of M. Trick [18, 19], see Methods; the algorithm is better suited to large graphs than exact methods such as of DSATUR [20].

Fig 2 summarizes the approach used to globally resolve the contact conflicts: defining the graph of conflicts, the coloring approach and the interpretation of the colors. We applied the coloring algorithm to the conflict graphs generated from the yeast HiC dataset, with several values of the cutoff parameter a between 1.3 and 2.0. The results agree with our expectation that the large numbers of conflicts can be reconciled with only several homogeneous conformations contained in the sample.

The results are summarized in Table 2, and an example coloring of a subset of the interaction graph for a = 2.0 is shown in Fig 2 (right panel).

**Table 2:** Minimum numbers of colors (genome states) needed to reconcile the yeast HiC data, depending on the threshold of *a* in conflict definition (Eq 1).

a	Pairs in conflicts	N colors	Locus-pairs with conflicting interactions of at most this many colors									
			1	2	3	4	5	6	7	8	9	10
1.3	86781	10	21702	40809	58248	76832	83164	85957	86462	86703	86763	86781
1.4	62093	8	17610	32010	48377	57581	61307	61971	62070	62093		
1.7	30085	5	10255	21636	28862	30050	30085					
2.0	16551	4	7057	14734	16528	16551						
Rand	22006	22	7420	10863	13248	15096	16652	17959	19053	19909	20547	21004

The result confirms that the HiC data of ([11]) can indeed be reconciled as a product of a small number of subpopulations in the experimental sample. Specifically, using the threshold *a* = 2.0, we can demonstrate that as few as 4 subpopulations of cells are sufficient to explain all the 30953 conflicts in the data. Moreover, even for low *a* there are only very few loci whose interactions require more than 5 colors. This observation may suggest that the majority of the conflicts are caused by the presence of only 3–4 rigid, homogeneous subpopulations and the remaining conflicts are only a very small fraction of the total and are caused by some kind of experimental artifact, possibly sequencing bias. It is important to note, that by modeling the sample as a mixture of only a few populations, we reduce the number of geometric conflicts from hundreds of thousands to zero, which constitutes an obvious improvement over previous approaches to infer the global geometry of the genome.

To assess how significant are the differences between the respective postulated conformational states, we list the numbers of conflicting interactions between each pair of states, in the case of a = 2.0 and four states needed to reconcile the entire dataset. The results in Table 3 show that the differences are large for each pair of states 1–3, suggesting a global reconfiguration of the genome into three main states.

**Table 3:** Numbers of conflicts between each pair of states for a = 2.0.

State	2	3	4
**1**	22869	4241	83
**2**		3662	46
**3**			51

Finally, we demonstrate that the distribution properties of the network of interactions between the states (“colors”) is not consistent with a random distribution of conflicts. To this end, we randomized the assignment of edges in the conflicts graph (to represent random conflicts between existing 3C interactions). The result is drastically different (see bottom row of Table 2), suggesting that the conflicts do not arise from measurement errors, but from actual states of the shape of the genome.

**Fig 3:**
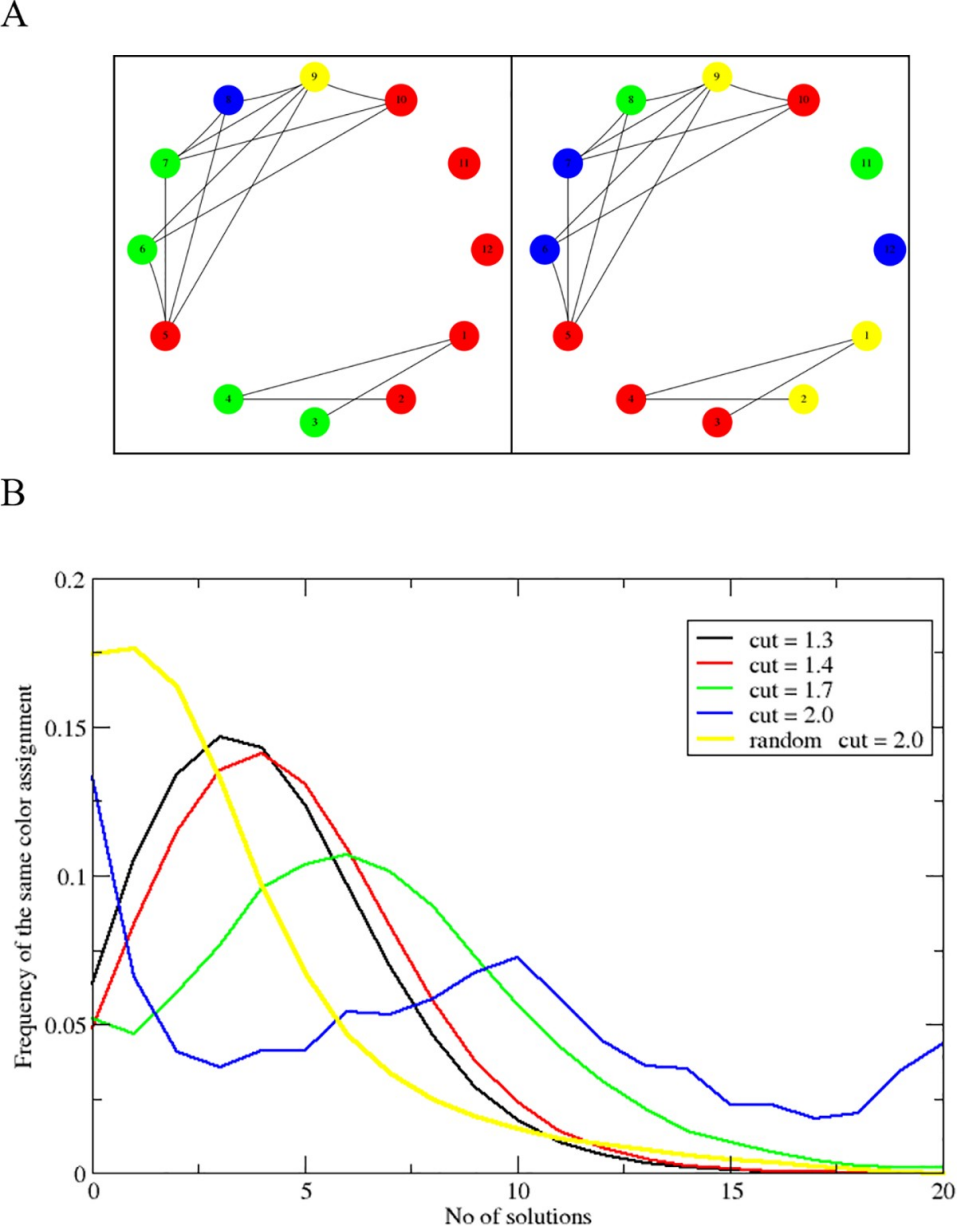
Alternative coloring solutions. A: Illustration of different colorings of the same conflict graph. B: Consistency between alternative coloring solutions in yeast HiC data. The figure shows the histograms of the number of similar color assignments among 20 possible coloring solutions. The histogram for the HiC contact conflict graph shows reasonable stability as evident by the fact that the histogram has its peak away from zero (a = 1.3,1.4, and 1.7). On the other hand, a random graph has its peak at zero which means that the coloring solution is less stable than real data. Finally, we see that the graph loses its coupling at high a, causing the peak at zero for a = 2.

In most cases, there are multiple solutions for coloring a graph using the minimal number of colors (See Fig 3A). To test the stability, or similarity between alternative solutions, we ran the coloring algorithm 20 times, randomly re-ordering the list of graph nodes on its input. In each case, the algorithm produced the same number of colors, but the assignments of colors to interactions was different in each solution. To test the consistency of coloring (or assess the similarity between the results from different random seeds), we assessed how many pairs of interactions that were in the same color in one solution remained of the same color in other solutions. The results are summarized in Fig 3B and in Supplementary S1 Table, and demonstrate that although not identical, the solutions have a significant degree of similarity, distinguishing them from random assignment of states. In other words, there is a significant global coordination between contacts across the entire genome; the identity of interactions attributable to each state is genuine.

It should also be noted that our result (small number of conformations needed to explain all the conflicts in the data) does *not* point to experimental artifacts as the main source of the violations of triangle inequality. Although significant contribution from experimental errors, especially sequencing bias, is not ruled out, it can be argued that such errors would lead to a very different graph representing the conflicts in the contact network. Specifically, as suggested by [21] and numerous subsequent studies, the histogram of relative sequencing coverage (or sequencing bias) has a positive skewness, with a small fraction of loci producing very high relative coverage. In a HiC experiment, such positive skewness is expected to result in a small number of “hubs”–highly connected loci whose interactors do not have interactors other than with the hub. Such hubs will correspond to large cliques in their line graphs (conflict graphs), and will thus require a large number of colors (states) to reconcile the data, a situation completely different from our analysis of the HiC data of Duan et al.

### Functional characterization of the sub-populations

**Fig 4:**
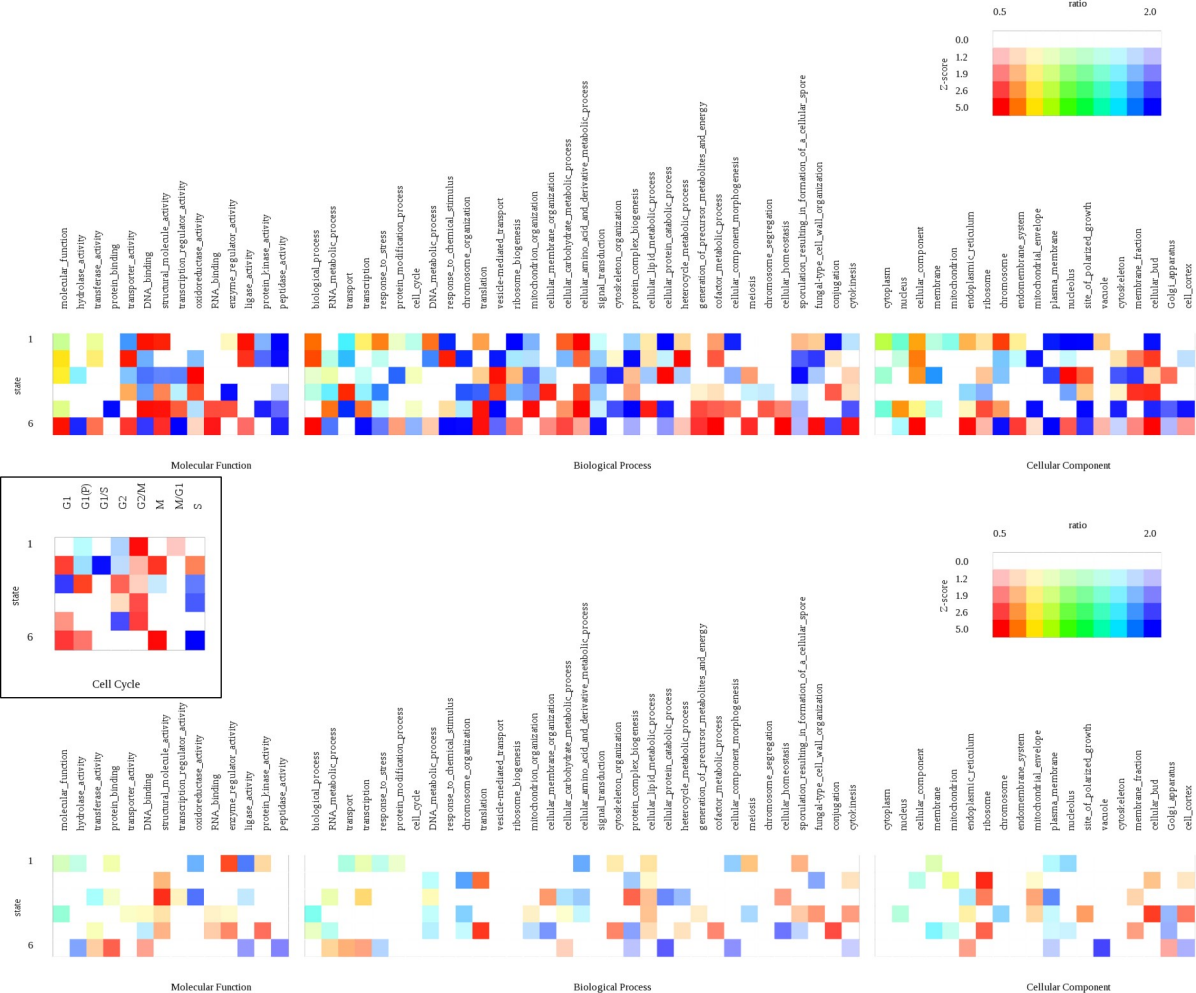
Analysis of functional annotation (significant GO terms) of genes whose loci interact in each of 5 inferred conformational states of the genome (top), compared to similar analysis for locus pairs with randomly assigned states (bottom). Inset: enrichments of genes associated with specific phases of the cell cycle.

We have demonstrated that the apparent conflicts observer in HiC data can be explained by a small number of homogeneous subpopulations of cells contained in the sample. Statistical considerations presented above suggest that at least to some extent the subpopulations are genuine and reflect biologically relevant coordination between contacts in different parts of the genome. It has been demonstrated that a correspondence exists between spatial organization of the yeast genome and transcriptional regulation of genes: genes in interacting genomic loci are often coexpressed and share the same functional annotations [22]; the dependence also exists in other species [23–25]. Moreover, global change in chromatin conformation has been associated with transition between functional states of the cell, such as quiescence [26]. If the chromatin states of cell subpopulations are indeed real, they should be subject to evolutionary pressure, and thus functionally relevant. In order to understand the biological significance of these states, we analyzed the distribution of groups of genes with different functions among these states. To confirm the functional role of the different conformational states we calculated the enrichment of different GO-slim term within the 6 states and compared with that of randomly created states. As it can be seen from Fig 4, many groups of genes tend to behave differently in different conformational states confirming the dynamical role that the genome conformation plays in regulating the functions of genes. The figure also shows that the number of significantly depleted or enriched groups of genes in the 6 conformational states is much higher than that of 6 random states confirming the nonrandom nature of these 6 genome confirmations.

The same dynamic behavior of these conformational states is also observed in different cell cycle phases [27] and metabolic cycle clusters [28]. The data for the cell cycles is shown in the inset of Fig 4. The results suggest that, especially during the cell cycle, specific inferred conformational states of the genome bring together genes that are active in specific phases of the cycle.

## Conclusions

In this work, we have presented a new hypothesis to explain the observed statistical characteristics of chromatin contacts as measured by HiC experiments. Using a graph-theoretic computational approach, we demonstrate that all the apparent geometric inconsistencies can be reconciled by a mixture of states present in the experimental sample, each state associated with a globally different configuration of chromatin. It has to be noted that the dynamic nature of chromatin may be a result of a combination of genome-wide reconfiguration with other effects, like local stochastic motions. Moreover, experimental errors can also affect the way an ensemble of configurations will present itself in HiC data.

However, while we do not claim that the global conformation of chromatin is always in one of a small number of discrete states, we demonstrate that such model will be sufficient to reconcile the HiC measurements event if no other phenomena were present to explain the inconsistencies in the geometry of the problem.

If our hypothesis of small number of discrete configurations is correct and the phenomenon is the primary source of the apparent violation of the triangle inequality, our method would constitute the first technique to globally reconcile geometric inconsistencies observed in HiC data. Our approach could then lead to improved understanding of the dynamics of genome conformations and its interplay with transcriptional programs. We have designed a graph-theoretic approach to determine the number of states needed to reconcile the data. Our implementation of the approach allows to find example solutions for genomic loci and their interaction that is limited to a specific conformational state of the genome. By applying the method to a yeast HiC dataset, we have shown that HiC data can be interpreted as a mixture of a small number of homogeneous states (we do not conclude that it *is* such a mixture, but that such interpretation is consistent with the data). Coordination between pairs of loci interacting in these states suggests the states may be biologically relevant/significant. The biological significance is supported by analysis of the functional categories of genes interacting in each thus defined state. The functional annotations are non-random, which is consistent with the hypothesis that the mixture of states actually exists, and that different states may be associated with the cells executing specific transcriptional programs. The possibility of global, coordinated changes in chromatin conformation may have consequences not only for transcriptional programming, but also for genome stability and DNA repair [29]; such interplay may be confirmed by correlating the inferred states with DNA damage patterns studied at high resolution [30, 31]. Finally, the approach may be generalized to use also with diploid nuclei and applied to studying conformation dynamics of the human genome. Such prospect is very promising, especially in light of the recent evidence (supported by computer simulations) suggesting that also in metazoans distinct cell populations may exist that realize specific structures of their topologically associated domains, and corresponding subpopulation-specific pattern of transcriptional activation of genes, see e.g. [16, 32, 33]. It is also important to note that our graph-coloring approach is not limited to distances and conflicts defined by Eqs (2) and (3) but it is a general framework that can be used with any method of assessing the distance and its error from the number of reads in the HiC dataset.

Properites of individual cells in a population may be also inferred from single-cell HiC experiments, but single-cell HiC only allows one link per restriction site, which is a serious limitation of the method used in kilobase-scale for an individual cell. For the same reason, geometric inconsistencies cannot arise in single-cell HiC, because once an interaction “A-B” is observed in a single cell, the interactions “B-C” or “A-C” cannot be probed–as the DNA associated with the restriction sites A and B is already used in the ligated A-B interaction. Finally, in a single cell, a link either is or isn’t observed, so it is not possible to distinguish between stronger (closer) and weaker (more distant) interactions.

On the other hand, a large collection of single-cell HiC datasets will allow to assess the joint probability distribution of interactions between different pairs of loci, from which information about global conformational states could be inferred. In this respect, one HiC experiment with our graph-coloring analysis may be thought of being equivalent to a whole series of single-cell HiC experiments. Our simple, computational approach could provide an alternative to technically challenging single-cell chromatin capture experiments, a direct comparison of the graph-coloring approach with multiple single-cell experiments will be a very interesting approach to experimental validation of the biological relevance of our method.

## Methods

### Identifying conflicts in HiC contacts

The HiC (referred to as “4C” by [6]) yeast dataset consists of a list of genomic loci of different captured DNA fragments. The genomic positions for the two ends of each fragment are provided in addition to the count frequency of each fragment. The count frequency for a captured fragment represents the contact probability between the two sites connected by the fragment. The frequency is also related to the distance between contacted sites. The contact frequency can be converted into a distance assuming that this frequency is equivalent to that of a polymer packing problem, yielding an approximately inversely proportional dependence. Thus, for each observed contact, we can estimate the Euclidean distance between the two ends of that contact.

In order to investigate whether the HiC contact data for yeast represents a static conformation or multiple conformational states, we look for conflicts between triplets of interacted sites. In the data under consideration, there are many triplets of contacted sites that would form triangles. Assuming one static conformation of the yeast genome, the observed distances in each triangle should obey the triangle inequality. On the other hand, the presence of different conformational states in the data will manifest itself in triplets that violate the triangle inequality (Fig 1). Those triplets that violate triangle inequality represent the contact conflicts that we seek to analyze. The conflicts are characterized by impossible triangles where one of the edges is larger than the total length of the other two or that one of the edges of the triangle is missing. The missing contacts are assigned a frequency of 4 (The minimum reported frequency is 5).

### Resolving conflicts in HiC contacts

The list of all observed conflicts is used to create a dual graph G = (V, E) where each vertex in V represents a contact (two interacting sites). Two vertices are connected by an edge in E if they are in conflict (Fig 2). In order to resolve these conflicts, we utilize the graph coloring techniques from the graph theory. The graph coloring scheme used here is “vertex coloring” where two connected vertices are colored (labeled) differently. This means that two conflicted contacts will be colored differently and thus belong to different colors (or states). A graph coloring algorithm will find the minimum number of colors for a graph and color the vertices according to this minimum. The graph coloring algorithm used here is a modified heuristic argument of [18, 19]. We used Michael Trick’s modified heuristic approach [18, 19] rather than other popular, exact methods such as of DSATUR [20], since it is significantly faster while producing equivalent results. The principle of the algorithm is summarized below:

- Find the maximum clique in the graph and coloring it with the UB colors.
- Order the adjacent nodes based on the degree of saturation (number of different adjacent colors).
- The first adjacent node with the highest degree of saturation is colored using one of the UB colors.
  ○ Loop over the nodes using the degree of saturation order and color sequentially until the graph is completely colored.
- If the number of colors needed is higher than UB then go back to step 3 using the next available color from the UB colors.”

Running the coloring code of [19] with the large graphs is memory-intensive. For this reason, we optimized the code by modifying the memory management and replacing 4-byte integers with 1-byte where adequate; the resulting implementation was able to process the graph for the cutoff factor a = 1.3 using less than 26GB of RAM. The algorithm has been validated to produce correct coloring results on randomly colored graphs (Supplementary S2 Table). Fig 2 summarizes the scheme used to resolve the contact conflicts.

## Supporting information

S1 Table Stability of color assignment in HiC conflict data. See discussion in text and Fig 3B.
(DOCX)

S2 Table Validation of the coloring algorithm. The program correctly reconstructs coloring of a random graph 96%-100% of the time in situations similar to the HiC conflict graphs.
(DOCX)
