# Supplementary material for: Maximum parsimony interpretation of chromatin capture experiments
Source: PLoS One. 2019 Nov 25;14(11):e0225578. doi: 10.1371/journal.pone.0225578 (PMC6876987; doi:10.1371/journal.pone.0225578)
Supplement: S1 Table — See discussion in text and Fig 3B. (DOCX) [file pone.0225578.s001.docx]

|  | Number of pairs with the same color assignment | | | | |
| --- | --- | --- | --- | --- | --- |
| Solution # | a=1.3 | a=1.4 | a=1.7 | a=2.0 | Random |
| 0 | 238878052 | 93460001 | 23453359 | 18242236 | 42267535 |
| 1 | 397776574 | 161328807 | 21195795 | 9013357 | 42778146 |
| 2 | 503862279 | 222015860 | 27417448 | 5552161 | 39635710 |
| 3 | 552315538 | 261383161 | 34586402 | 4895529 | 32011105 |
| 4 | 537259171 | 271862909 | 43271838 | 5646532 | 23346736 |
| 5 | 466791292 | 252446561 | 47014258 | 5626454 | 16261864 |
| 6 | 366074091 | 210844233 | 48399118 | 7446157 | 11343045 |
| 7 | 261922684 | 160309454 | 45976472 | 7306510 | 8113820 |
| 8 | 173936756 | 112168651 | 40543816 | 7977809 | 6045717 |
| 9 | 109283848 | 73380652 | 32921477 | 9251226 | 4623419 |
| 10 | 66676896 | 45692366 | 25598583 | 9922493 | 3631857 |
| 11 | 39429643 | 27459352 | 19204856 | 8042986 | 2900851 |
| 12 | 23090272 | 16041795 | 14004912 | 6092368 | 2380575 |
| 13 | 12872171 | 9088297 | 9670137 | 4918157 | 1946667 |
| 14 | 6930009 | 5030586 | 6439257 | 4796676 | 1547835 |
| 15 | 3597478 | 2667218 | 4633138 | 3160289 | 1173116 |
| 16 | 1720303 | 1334470 | 3216547 | 3133357 | 879215 |
| 17 | 743551 | 670758 | 1955816 | 2511695 | 604488 |
| 18 | 346099 | 294454 | 1180984 | 2768869 | 370484 |
| 19 | 121769 | 124352 | 905657 | 4699983 | 191483 |
| 20 | 237227 | 135341 | 948700 | 5954681 | 67347 |

**Supplementary Table S1**: Stability of color assignment in HiC conflict data. See discussion in text and Figure 3b.
