# Supplementary material for: Maximum parsimony interpretation of chromatin capture experiments
Source: PLoS One. 2019 Nov 25;14(11):e0225578. doi: 10.1371/journal.pone.0225578 (PMC6876987; doi:10.1371/journal.pone.0225578)
Supplement: S2 Table — The program correctly reconstructs coloring of a random graph 96%-100% of the time in situations similar to the HiC conflict graphs. (DOCX) [file pone.0225578.s002.docx]

| # of state | State 1 | State 2 | State 3 |
| --- | --- | --- | --- |
| 2 low density | 100% | 100% |  |
| 2 high density | 100% | 100% |  |
| 2 low density^b^ | 100% | 100% |  |
| 2 high density^b^ | 100% | 100% |  |
| 3 low density | 100% | 100% | 100% |
| 3 high density^c^ | 96% | 100% | 100% |

1. States: 1000, 500
2. States: 2000, 1000
3. States: 2000, 1000, 1000

**Supplementary Table S2**. Validation of the coloring algorithm. The program correctly reconstructs coloring of a random graph 96%-100% of the time in situations similar to the HiC conflict graphs.
